# Design and Fabrication of Compact, Multiband, High Gain, High Isolation, Metamaterial-Based MIMO Antennas for Wireless Communication Systems

**DOI:** 10.3390/mi14020357

**Published:** 2023-01-31

**Authors:** Ammar Armghan, Shobhit K. Patel, Sunil Lavadiya, Salman Qamar, Meshari Alsharari, Malek G. Daher, Ayman A. Althuwayb, Fayadh Alenezi, Khaled Aliqab

**Affiliations:** 1 Department of Electrical Engineering, College of Engineering, Jouf University, Sakaka 72388, Saudi Arabia; 2 Department of Computer Engineering, Marwadi University, Rajkot 360003, India; 3 Department of Information and Communication Technology, Marwadi University, Rajkot 360003, India; 4 Department of Electrical Engineering, Qurtuba University of Science and IT, Dera Ismail Khan 29050, Pakistan; 5 Physics Department, Islamic University of Gaza, Gaza P.O. Box 108, Palestine

**Keywords:** antenna, MIMO, directivity, gain, compact, wireless communication systems

## Abstract

We proposed a novel approach based on a complementary split-ring resonator metamaterial in a two-port MIMO antenna, giving high gain, multiband results with miniature size. We have also analyzed a circular disk metasurface design. The designs are also defected using ground structure by reducing the width of the ground plane to 8 mm and etching all other parts of the ground plane. The electric length of the proposed design is 0.5λ × 0.35λ × 0.02λ. The design results are also investigated for a different variation of complementary split-ring resonator ring sizes. The inner and outer ring diameters are varied to find the optimized solution for enhanced output performance parameters. Good isolation is also achieved for both bands. The gain and directivity results are also presented. The results are compared for isolation, gain, structure size, and the number of ports. The compact, multiband, high gain and high isolation design can apply to WiMAX, WLAN, and satellite communication applications.

## 1. Introduction

Antenna design has changed extensively over the past couple of decades. The antenna size has reduced to a great extent in this time. The antenna designs have shifted from bulkier horn antennas to small microstrip antennas. However, there is still scope for improvement, and researchers are working on achieving smaller antennas to accommodate small handheld portable devices. The antenna’s reduction in size and weight can be handy in mobile devices as it can create a space for new applications to be accommodated in portable devices. The size reduction and high gain are the two critical parameters that can apply to many applications if achieved together. The metamaterial can be used to accomplish these essential parameters in multiple-input multiple-output (MIMO) antennas. 

Comparing a MIMO system to a single-input single-output (SISO) system, the former is superior in terms of gain, bandwidth, channel capacity, and diversity performance. As a MIMO system does not have to retransmit information repeatedly, the data throughput is significantly increased. An additional benefit of a MIMO system in a dense scattering environment is a higher data rate with less channel capacity loss. This is achieved without increasing the transmitted power or the spectrum used [1]. A combination of different methods is used to improve MIMO performance. Short-range wireless local area networks (WLANs) are one of several wireless communications applications that use antennas. Researchers are keen on WLAN MIMO systems because of their potential to improve the quality of wireless connections between various electronic gadgets. WLAN networks rely on base stations, sometimes called access points, to link wireless clients to the network [2].

Metamaterials are artificial materials that can be incorporated into antennas to improve their parameters such as gain, size, bandwidth, etc. Metamaterial antennas can enhance the bandwidth and gain by incorporating metamaterial elements in antennas [3]. Metamaterial components can be added to the dipole antenna to reduce its size and achieve reconfiguration [4]. Gain and bandwidth enhancement are achieved using Radar cross-section (RCS) reduction with a resonating antenna with a metamaterial superstrate [5]. Pattern reconfiguration and frequency tunability is achieved using metamaterial antennas [6]. Liquid metamaterial is a new concept, and the metamaterial antenna based on this new concept is implemented in [7]. Multiband antenna design is presented with metamaterial superstrates. Metamaterial superstrates are formed with liquid split ring resonator (SRR) and copper SRR [8]. Metamaterials can also be added to the MIMO antenna to improve its parameters. Metamaterial MIMO antennas can be used to enhance parameters such as isolation, gain, bandwidth, and size. The size and gain of the MIMO antenna can be improved with a metamaterial superstrate [9]. Antenna diversity may be accomplished using various approaches, including polarization, space, and pattern diversities, or by combining these and other methods. One or more of these many diversity schemes may be used to achieve diversity gain, depending on various considerations, including space, environment, and interferences [10].

Ultrawideband (UWB) MIMO technology combines UWB and MIMO technologies to maximize the benefits of each, allowing for a faster data transmission rate and increased channel capacity while mitigating multipath fading. Scholars have also found UWB MIMO antennas to be an exciting area of study in recent years. The main issue with UWB MIMO antennas is their poor coupling. Due to the high speed of today’s mobile terminals, mobile communications and wireless devices are shrinking and converging. As UWB MIMO antennas continue to shrink in size, electromagnetic interference will become more severe due to the decreased spacing between antenna components and increased strength of mutual interaction [11]. Higher coupling between antennas may be achieved by using metamaterial structures, which concentrate electromagnetic fields and currents around antenna structures rather than distributing them over the antenna ground. Antenna and passive circuit applications may benefit from metamaterial technology’s ability to shrink circuit size while maintaining or improving performance. Due to this, the circuit size does not change with the operating frequency. Hence it may be drastically shrunk to fit in a little space. Due to the negative permeability feature of an interdigital split ring resonator, a MIMO antenna with increased isolation for the Long-Term Evolution (LTE) frequency range has been developed using SRR [12]. The article proposes an integrated massive MIMO antenna system loaded with metamaterial for fifth-generation (5G) applications. The metamaterial approach aids in realizing DNG characteristics via the use of a complementary SRR (CSRR) structure, a wide epsilon negative metamaterial with near-zero refractive index properties, and more than 1 GHz bandwidth [13]. The metamaterial is printed on top of dielectric resonators to increase isolation, which moves the most vital coupling forces away. Field distributions are altered as the metamaterial structure interacts with the EM fields, resulting in decreased linked areas. The suggested antenna construction is simple and compact [14]. A compact broadband planar monopole MIMO antenna is used for long-term advanced evolution. Using two innovative broadband metamaterial split-ring resonator units, the mutual coupling of the MIMO antenna is lowered, while the antenna’s impedance bandwidth is increased [15].

Effective efficiency at mm wavelengths requires substantial suppression of the substrate modes. These modes may be attenuated in several ways, including keeping the substrate at a constant thickness, keeping the element spacing in arrays at a constant value of k0, and employing the substrate-based waveguide approach. It grounds the surface modes using metallic vias [16]. A band-notched characteristic with wideband omnidirectional dielectric resonator antenna (DRA) is used to generate an omnidirectional radiation pattern, and different modes of a rectangular DR are activated using a laminated feed structure [17]. The 5G communication can be implemented with an antenna designed with metamaterials for a 28 GHz frequency band [18]. Over the last several years, there has been a lot of focus on improving the isolation between many antennas in compact terminals. Isolation is an important parameter of the MIMO antenna, and high isolation is required between the ports. The high isolation can be achieved with a metamaterial-inspired MIMO antenna design [19]. The use of decoupling structures in space and linking decoupling networks to the feeding ports of antennas. A line that has been neutralized, defective ground constructions, electromagnetic band gaps, and other decoupling structures in space are intended to affect antennas’ emf fields or ionic currents. Distributed, lumped, and resonator networks are examples of decoupling networks that separate ports by modifying feeding signals [20,21]. The oval MIMO antenna has a circular radiator and two parallel protruding stubs with open ends; the concentric rings include oval slots (CROS). Circularly polarized (CP) radiation is generated by strategically placing three oval slots in the concentric rings, two of which are exposed to the outside [22]. The monopole antenna’s radiating element has been loaded with numerous narrow slots and multiple slotted stubs to produce multiband characteristics (MSS) [23]. The stepped DR enhances isolation in the higher frequency range, while a modified neutralization line realized using a quarter wavelength square patch accomplishes the same goal in the lower frequency range. The gain and radiation properties of the stepped DR and the artificial magnetic conductors (AMC) reflector are enhanced by their broadside radiating modes and dual artificial magnetic conductors and PEC bands, respectively [24].

A MIMO antenna with a four-port design is applied for wireless networking applications [25]. Furthermore, the reduction in size with ultra-wideband response is achieved with metamaterial loading in the MIMO antenna design [26,27]. Recently, a team of researchers has improved isolation with metamaterial loading in the MIMO antenna [28]. Pattern diversity and polarization are helpful methods for reducing the correlation among antenna far fields without increasing the size. CP antennas provide benefits, including multipath fading, lower signal attenuation, and absorption losses [29]. Due to less space and dimension when arranging antennas, many MIMO antennas for WLAN are proposed in the current era. Most research focuses on approaches to provide adequate isolation and reduce mutual coupling [30].

Due to the constrained space, a small antenna with strong isolation is recommended for MIMO communication. Investigations were undertaken on the MIMO antenna’s impact from the mutual coupling. The reduction in mutual coupling in a MIMO antenna has been researched using various strategies. To attain optimal magnetic conductors (MC), researchers have looked into several MC reduction approaches [31].

The truncated corners antenna and the parasitic element approach boost bandwidth. By combining two square patches separated by a diagonal slot and four parasitic components, we created a MIMO antenna with enhanced performance [32]. The MIMO antenna is constructed using periodic parasitic elements and truncated corner metallic radiators to boost isolation and the axial ratio [33]. Due to their small size, large peak gain, small envelope correlation coefficient (*ECC*), significant diversity gain, intense isolation, and wide operating frequency range, the presented MIMO antennas may be used in several WCOM applications.

The need for compact, multiband, high gain and high isolation MIMO antenna has led us to propose a complementary split ring resonator metamaterial-loaded two port MIMO antenna design. Our proposed design gives high isolation and high gain response with a small size and can be a good selection for different wireless communication systems. The design is showing better performance compared to other published works. Variation in various parameters is also carried out to find the optimized design. The structure is discussed in the next section, and the results and performance observing parameters are discussed after the concluding remarks have been given. 

## 2. Design and Modelling

**Figure 1:**
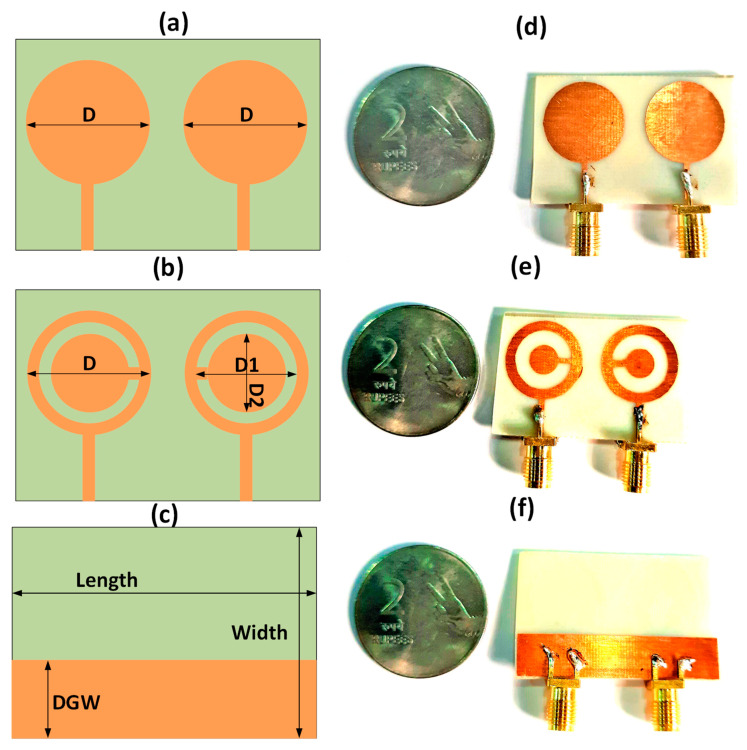
Proposed 2-port MIMO antenna. (**a**) Circular disk MIMO antenna design. (**b**) CSRR metamaterial-based MIMO antenna design. (**c**) Ground plane with the defected ground structure. The diameters D = 10 mm, D_1_ = 8 mm, and D_2_ = 6 mm. Length = 35 mm, width = 25 mm, and DGW = 8 mm. (**d**) Prototype top view of circular disk MIMO antenna. (**e**) Prototype top view of CSRR-based MIMO antenna. (**f**) Bottom view of prototype.

The new metasurface antenna design is constructed by etching the split ring resonator of a circular disk, as shown in Figure 1b,e. The simple circular disk design and complementary split ring resonator (CSRR) metasurface design are presented in Figure 1a,d. The ground plane is kept the same for both designs, as shown in Figure 1c,f. The ground plane is diffracted. The optimized width of the ground plane is 8 mm. The ring shape is also optimized with an outer ring of 8 mm and an inner ring of 6 mm. The length and width of the substrate are, respectively, 40 and 25 mm. The copper material is used in the resonator and ground plane. The electric length of the proposed design is 0.5λ × 0.35λ × 0.02λ. The substrate is made of FR-4 material with a thickness of 1.6 mm. The design dimensions of the presented work are represented in Table 1.

The primary design aim was to use inexpensive materials in the MIMO antenna’s construction. Therefore, the suggested design makes use of FR4 material. This material was chosen because it is easily accessible and has robust physical qualities that can resist the milling process. For the milling operation, high-velocity drill bits were manually scraped away from the exposed copper surface. Compared to the minimal resolution sizes of 0.1 mm and gaps of 0.6 mm necessary to manufacture the antenna, the milling machine’s precision was much superior. The milling operation was accomplished in under an hour as the antenna had simple geometries, and the bulk of the board was the ground plane, which did not need etching. Connecting the coaxial connections to the feed points on the antenna structure followed the milling of the MIMO antenna board. Coaxial connectors are the interface via which the antenna may be linked to third-party testing instruments. The antenna’s ground plane was bent to accommodate a coaxial line interface for a simple connection. The ground plane surface currents were assured to behave as predicted by the simulation by soldering at many points rather than only along the coaxial wire.

**Figure 2:**
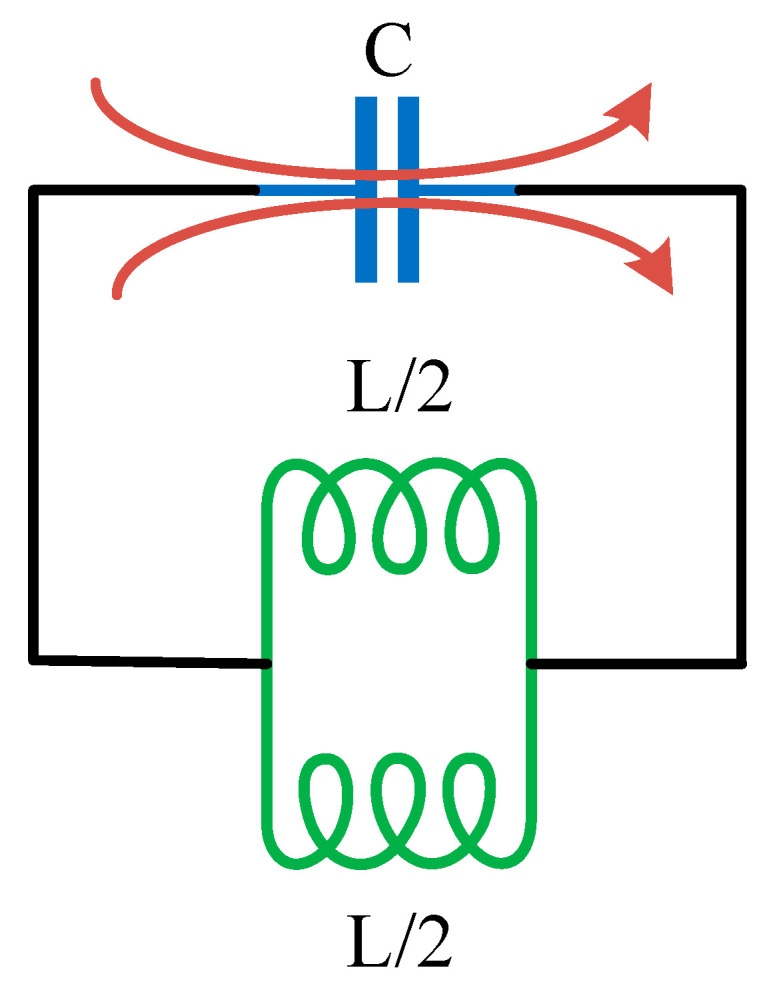
The equivalent model of the proposed CSRR circuit.

The electrical equivalent model of the presented design is represented in Figure 2. The capacitance represents the gap between the ring’s two terminals, and the structure’s wounded shape is represented by the two parallel connected indicators [34].

## 3. Results and Discussions

**Figure 3:**
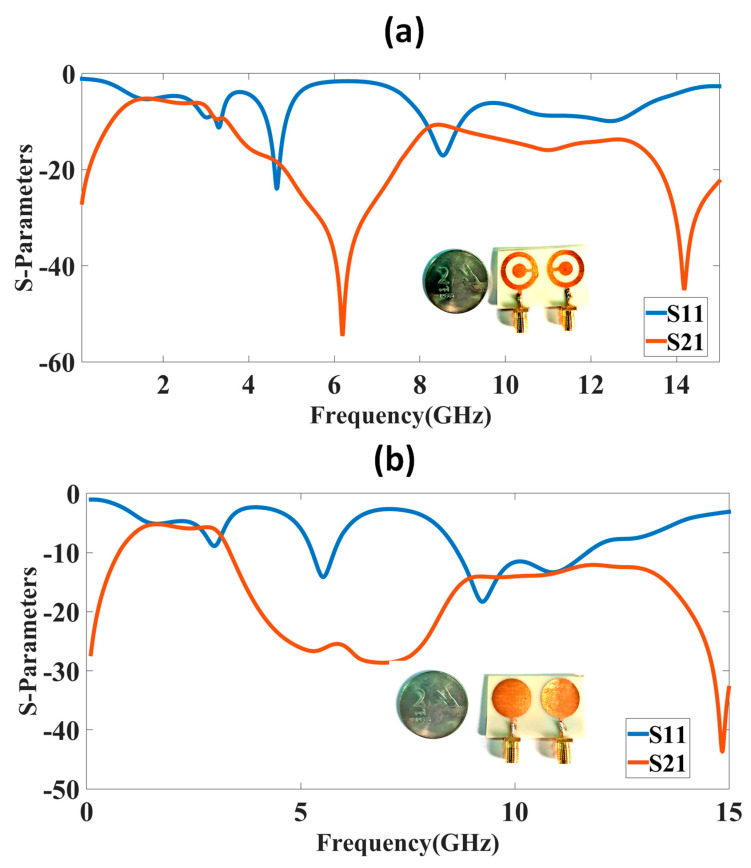
S-parameter results for two designs. (**a**) Complementary split-ring resonator metamaterial design. (**b**) Circular disk design. High isolation is available in complementary split-ring resonator metamaterial design with 55 dB. Two bands are available in design for C and X bands.

**Figure 4:**
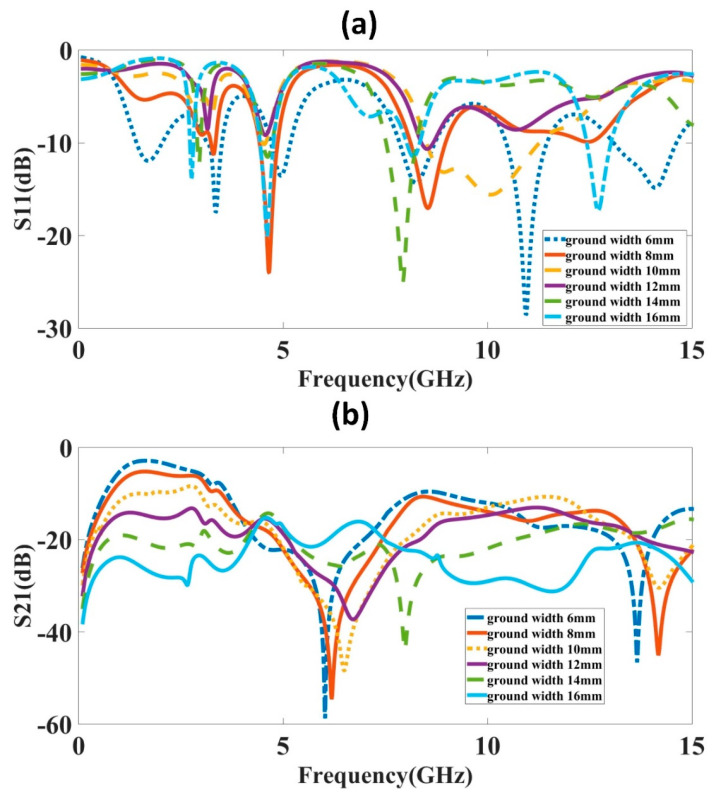
S-parameter results for different ground widths. Ground width is changed over 6 to 16 mm with a step size of 2 mm. (**a**) S_11_ (**b**) S_21_. The results show that for the ground width of 8 mm, both S_11_ and S_21_ have good results. The multiband response with good isolation is visible for 8 mm ground width.

**Figure 5:**
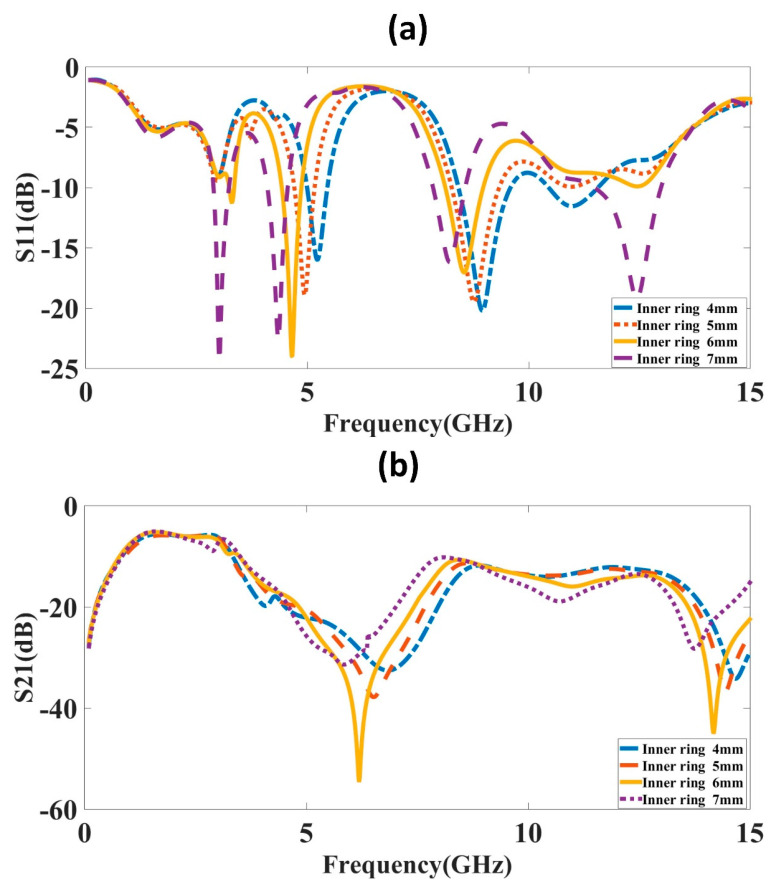
S-parameter results for different inner ring diameters. The inner ring diameter varies from 4 to 7 mm with a 1 mm step size. (**a**) S_11_ (**b**) S_21_. The results show that the inner ring diameter of 6 mm leads to good results. The multiband response with good isolation is visible for a 6 mm inner ring diameter.

**Figure 6:**
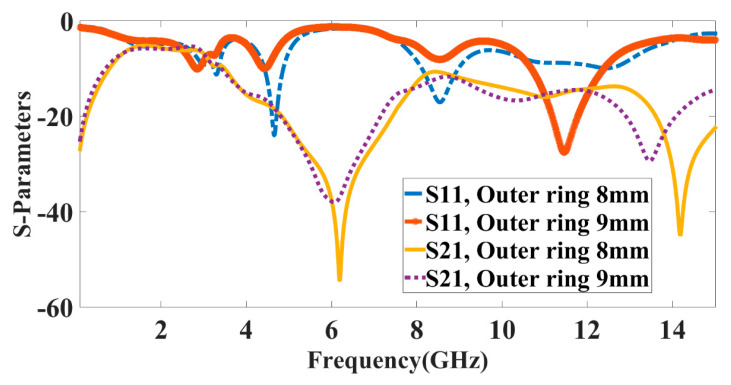
S-parameter results for different outer ring diameters. The inner ring diameter changed from 8 to 9 mm with a step size of 1 mm. Results show that an outer ring diameter of 8 mm shows good results. The multiband response with good isolation is visible for an 8 mm inner ring diameter.

**Figure 7:**
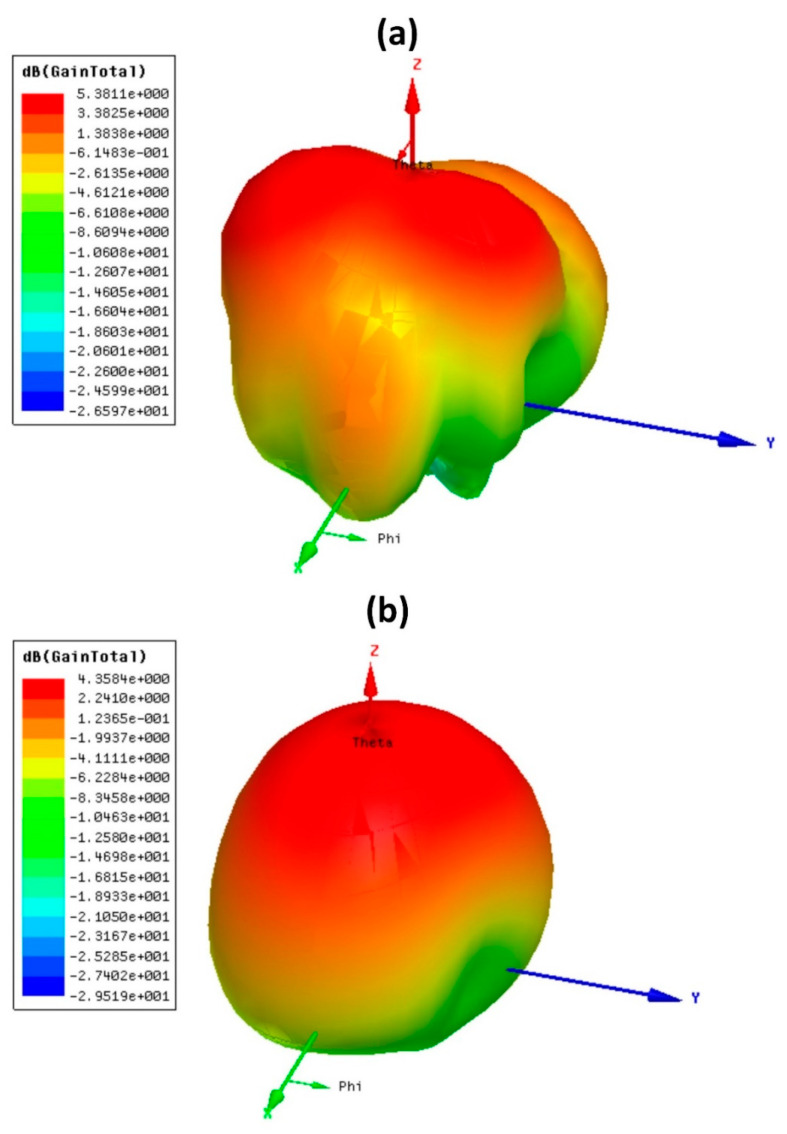
Gain polar plots for both designs. (**a**) Complementary SRR design and (**b**) circular disk design. The complementary split ring resonator design has a higher gain of 5.38 dB.

**Figure 8:**
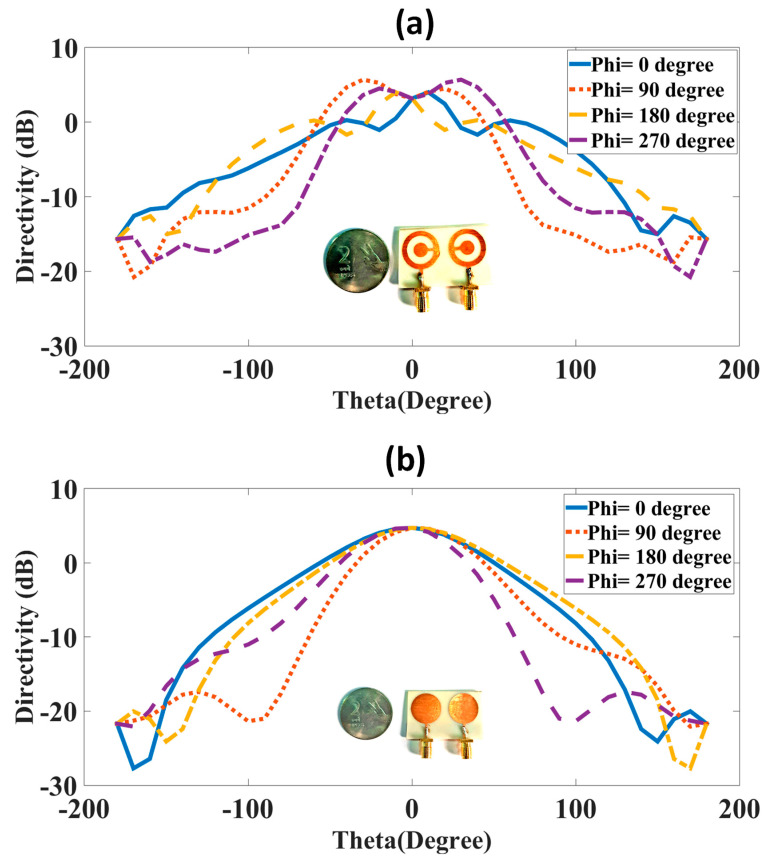
Directivity plot for both designs. (**a**) Complementary SRR design. (**b**) Circular disk design. The complementary split ring resonator design has higher directivity of 5.95 dB.

The structure presented in Figure 1 was simulated using the FEM-based HFSS tool, and the results are given in Figure 2, Figure 3, Figure 4, Figure 5, Figure 6, Figure 7 and Figure 8 for S-parameters, gain, and directivity. Initially, both designs were compared for their S-parameters, and the best design was optimized for different parameters. The S-parameter results are presented in Figure 3 for both techniques. The CSRR metasurface design results are presented in Figure 3a. The blue line is S11, and the orange line is S_21_. The results of the CSRR metasurface design show that it has two clear bands visible, with the first band having a frequency range of 4.42 to 4.87 GHz, corresponding to a bandwidth of 450 MHz. The second band has a frequency range of 8.11 to 8.98 GHz, corresponding to a bandwidth of 870 MHz and a maximum S_11_ of −25 dB. The highest isolation of 55 dB is achieved for the first band. The circular disk design results are shown in Figure 3b. The results of the circular disk design show that it has two clear bands visible, with the first band having a frequency range of 5.28 to 5.77 GHz, corresponding to the bandwidth of 500 MHz, but the highest S_11_ is only −14 dB with an isolation of 29 dB. The second band has a frequency range of 8.67 to 11.68 GHz, corresponding to a bandwidth of 3 GHz.

The ground plane width becomes defective by etching a piece of the ground plane width, which enhances the structure’s resonance characteristic. The result of dimension variations in the ground plane width is investigated in Figure 4. The variation is considered over 6 to 16 mm with a step size of 2 mm. The ground plane width variation from the plot clearly shows that the S_11_ results of the 8 mm ground plane width and 16 mm ground plane width have good values. Still, the isolation for both widths shows that the 16 mm results are in isolation of about 15 dB or less. Thus, we have selected an 8 mm ground plane width for these two MIMO antenna designs on the importance of the S-parameters and the number of bands and their applications.

The inner ring of the complementary split ring resonator is varied, and the variation in the results is observed in Figure 5. The variation in S-parameter results is observed from 4 to 7 mm with a 1 mm step size. The tuning waveform is visible for the change in the inner ring diameter. The first band shows good reflectance and isolation for the 6 mm inner ring diameter, so the optimized value for the inner ring diameter is kept at 6 mm.

The outer ring of the complementary split ring resonator is varied, and the variation in the results is observed in Figure 6. The variation in S-parameter results is observed from 8 and 9 mm. Similarly, the tuning is a waveform visible for the change in the outer ring diameter. The 8 mm outer ring radius shows better reflectance and isolation, as is visible from the blue line curve and yellow isolation curve. The red curve for the 9 mm outer ring shows less reflectance and only one band in the X band, while the 8 mm outer ring diameter gives two bands and good isolation for both bands.

The gain polar plot and directivity results were analyzed for both antenna designs, and their results are shown in Figure 7 and Figure 8. The 5.38 dB of gain results are presented in Figure 7 for the CSRR metasurface MIMO antenna. The circular disk design has the highest gain of 4.35 dB. The gain polar plot is oriented towards the positive z direction, which shows it has directional radiation. The directivity results of both antenna designs are presented in Figure 8. The directivity was analyzed for −180 to 180° theta values. The phi values were used with four degrees, and those were 0, 90, 180, and 270°. Two directivity peaks around 0° theta values are visible in the CSRR metasurface design. The circular disk design has a single peak at 0° theta value. Therefore, the directivity values are varied for the different phi values of the design.

**Figure 9:**
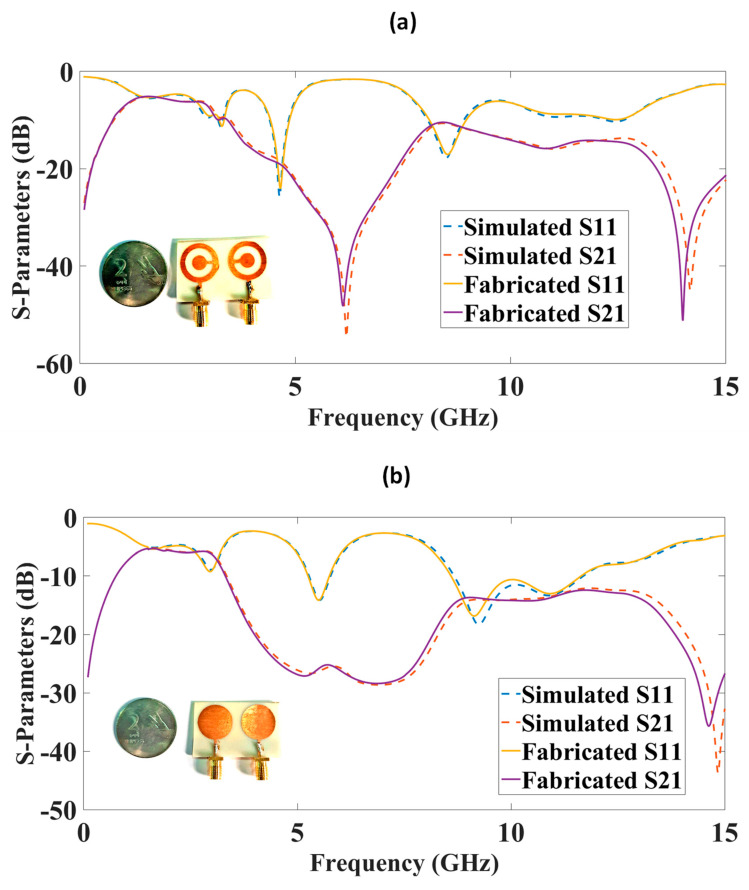
S-parameter comparison for the measured and simulated results. (**a**) Complementary split ring resonator design. (**b**) Circular disk MIMO antenna.

The S-parameters of both MIMO antenna designs were checked using a vector network analyzer. The results are presented in Figure 9. The agreements among S_11_ and S_21_ effects in the measured and simulated results were observed. The CSRR MIMO results are presented in Figure 9a, and the circular disk is shown in Figure 9b. The fabricated prototypes are presented as an inset in the figures.

**Figure 10:**
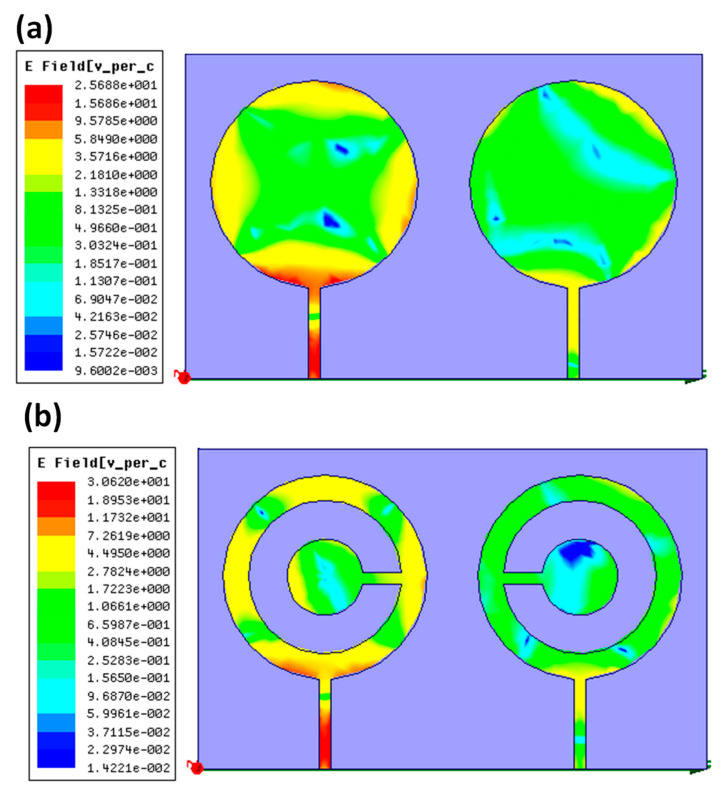
The surface charge distribution of the presented design. (**a**) The circular disk represents Efield of 2.56 × 10^1^ V/cm. (**b**) CSRR-based structure means an Efield of 3.062 × 10^1^ V/cm.

The charge distribution of the presented design is represented in the figure. The shape variation alters the surface charge distribution. Figure 10a shows the Efield of the circular disk-based patch is 2.56 × 10^1^ V/cm. Figure 10b shows the Efield of the CSRR-based structure is 3.062 × 10^1^ V/cm. A better surface charge was attained in the presented approach.

**Figure 11:**
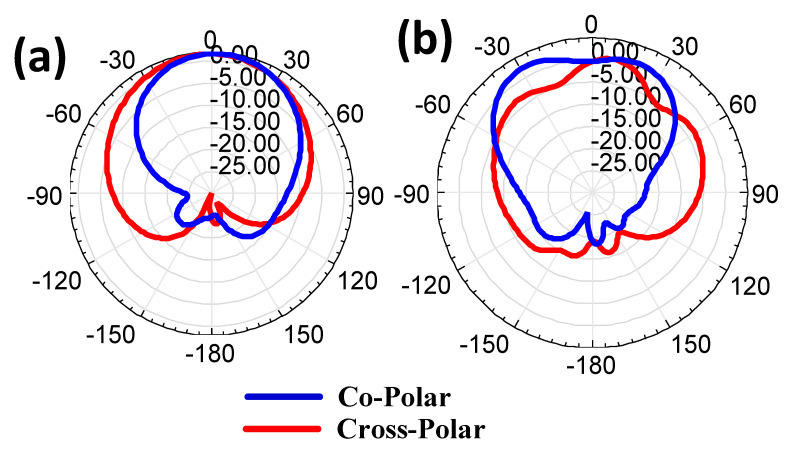
Radiation pattern of presented design structure. (**a**) Circular disk-shaped patch structure. (**b**) CSRR-shaped patch structure.

The presented design’s simulated copolar and cross-polar responses are shown in Figure 11. The copolar is used when the sender and receiver antenna elements are oriented in the same direction. The cross-polar is used when the sender and receiver antenna elements are orthogonal. The copolar and cross-polar plots for the circular disk-shaped and CSRR-shaped patch structures are represented in Figure 11a,b. The broader directivity is also observed for both design structures.

**Figure 12:**
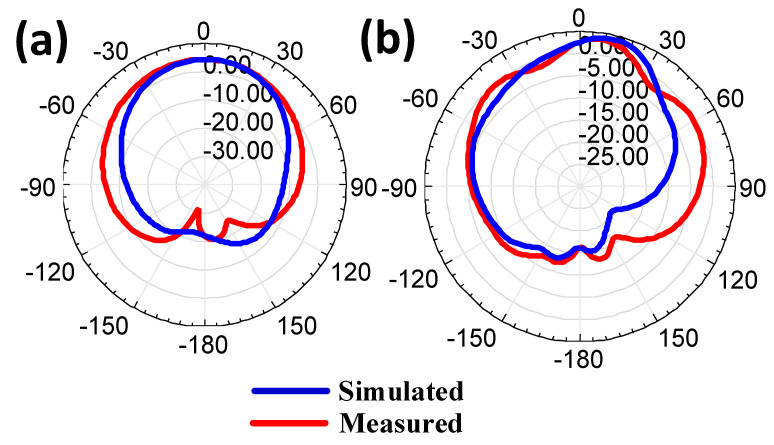
Antenna gain pattern of presented design structure. (**a**) Circular disk-shaped patch structure. (**b**) CSRR-shaped patch structure.

**Figure 13:**
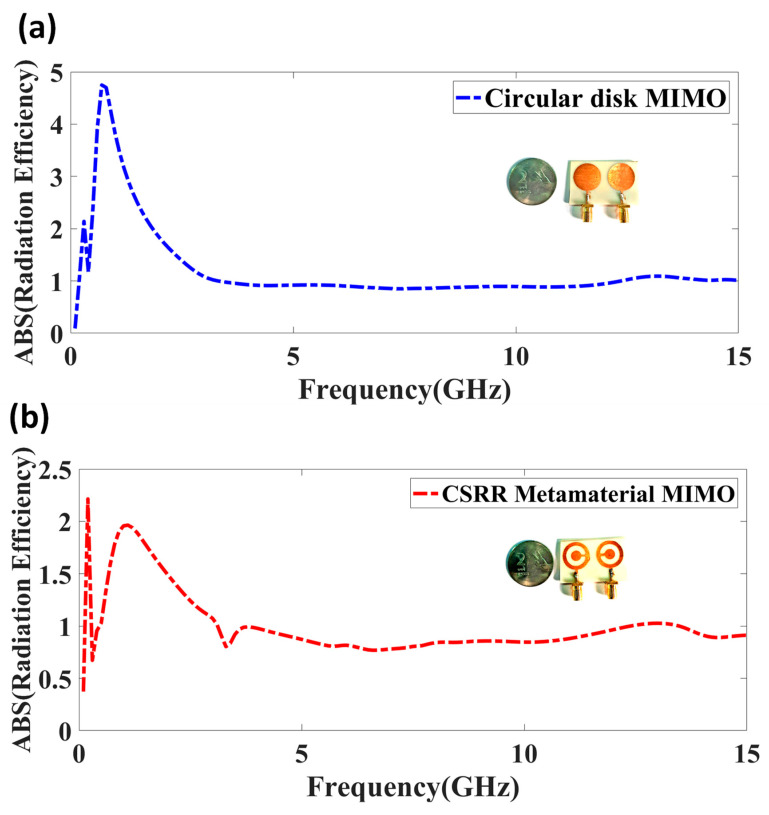
Absolute radiation efficiency of proposed design. (**a**) Circular disk-shaped MIMO antenna. (**b**) CSRR metamaterial-shaped MIMO antenna structure.

The proposed work’s simulated and measured antenna gain is represented in Figure 12. There is a good similarity observed between both results. The minor variation is regarded because the dimension variation, calibration issue, and fabrication glitches lead to slight variation among both results. The broader gain is attained in the CSRR metamaterial-loaded MIMO antenna structure. The performance of the antenna was analyzed based on the antenna efficiency. The absolute antenna efficiency of the proposed design is represented in Figure 13. The circular disk-shaped MIMO antenna and CSRR metamaterial- shaped MIMO antenna represent good radiation efficiency. The circular-shaped MIMO describes more than 0.92 of absolute radiation efficiency over the 4 to 15 GHz frequency span, and the CSRR-shaped MIMO antenna means more than 0.90 of absolute radiation efficiency over the 4.2 to 15 GHz frequency span.

## 4. MIMO Performance Parameters

In this section, we introduce the proposed MIMO antenna by computing its values for key crucial factors that define MIMO antenna systems, such as directional gain and equal-channel coupling (*DG* and *ECC*). This section provides examples of these parameters.

### 4.1. Envelope Correlation Coefficient (ECC)

Good MIMO performance is guaranteed by analyzing the antenna diversity presentation in terms of *ECC*. The envelope correlation coefficient (*ECC*) provides information on the degree of separation and correlation between various communication channels. The *ECC* of a multi-antenna system may be calculated by observing how one antenna’s emission pattern influences the others. The lower *ECC* is crucial for the robustness of the design. The *ECC* for a two-port MIMO antenna may be determined from the radiation pattern using Equation (1) and the transmittance and reflectance coefficient as per Equation (2).

(1) $$\rho e=\frac{{\left|\iint_{4\pi }\left[{\vec{F}}_{1}\left(\theta ,\phi \right)\times {\vec{F}}_{2}\left(\theta ,\phi \right)d\Omega \right]\right|}^{2}}{{\left|\iint_{4\pi }\left[{\vec{F}}_{1}\left(\theta ,\phi \right)\right]\right|}^{2}d\Omega {\left|\iint_{4\pi }\left[{\vec{F}}_{2}\left(\theta ,\phi \right)\right]\right|}^{2}d\Omega }$$

(2) $$\rho_{e}=\frac{{\left|S_{11}S_{12}+S_{22}S_{21}\right|}^{2}}{\left(1-\left|S_{11}^{2}+S_{21}^{2}\right|\right)\left(1-\left|S_{22}^{2}+S_{12}^{2}\right|\right)}$$

**Figure 14:**
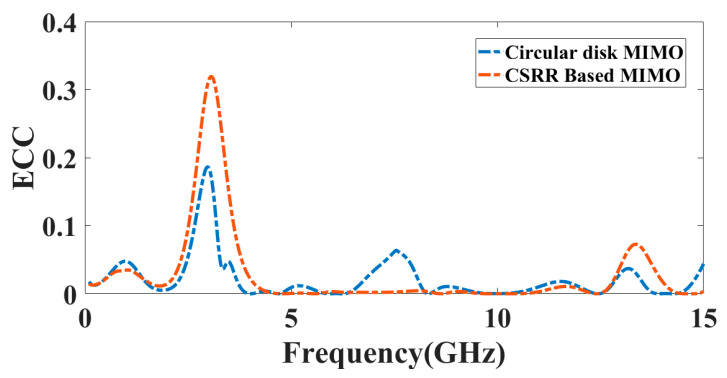
The *ECC* response of the proposed two-port MIMO antenna structure.

Using the far-field computation method, *ECC* is calculated. Here, ${\vec{F}}_{1}\left(\theta ,\phi \right)$ is the farfield pattern of the MIMO array during exciting the single port, and Ω signifies the solid angle. Practically, the value of *ECC* must be lower than 0.5 [35]. Figure 14 represents the *ECC* value near zero over a broad spectrum. The lower correlation among both antennae is observed based on the graph.

### 4.2. Diversity Gain (DG)

**Figure 15:**
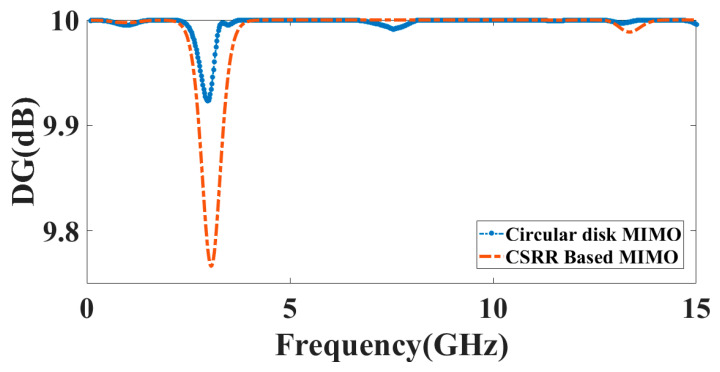
The diversity gain of the proposed antenna. The *DG* for circular disk MIMO structure is 9.93 dB and for the CSRR-based MIMO antenna is 9.77 dB.

The benefits of variety are often attained when transmitters receive numerous broadcast stream versions through diverse channel paths. Therefore, finding a diversity gain value close to 10 dB indicates a strong diversity performance. Diversity gain may be calculated using Equation (3) from the envelope correlation coefficient. Figure 15 represents the *DG* of the presented design.

(3) $$DG=10\times \sqrt{1-{\left(ECC\right)}^{2}}$$

### 4.3. Total Active Reflection Coefficient (TARC)

The *TARC* defines the degree of coupling and channel independence among different ports. To quantify the interaction between nearby cells in MIMO systems [36], use:

(4) $$TARC=-\sqrt{\frac{{\left(S_{11}+S_{12}\right)}^{2}+{\left(S_{21}+S_{22}\right)}^{2}}{2}}$$

**Figure 16:**
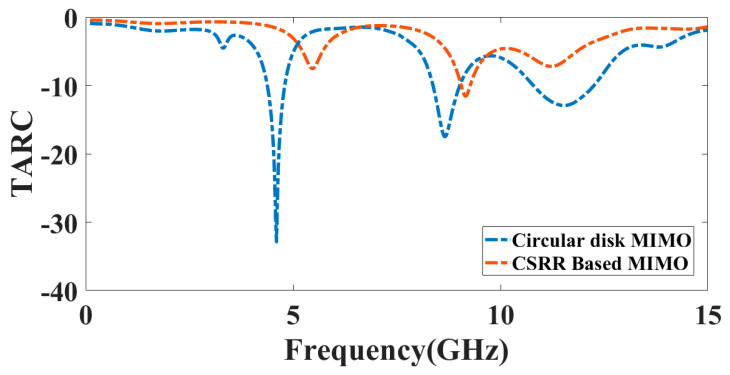
The *TARC* response of the presented circular disk and CSRR-based MIMO antenna structure.

The *TARC* value should be lower than 0 dB [36]. Figure 16 represents the reflectance of the *TARC* response for the circular disk MIMO observed at −32.89 dB at 4.6 GHz, −17.42 dB at 8.64 GHz, and −12.93 dB at 11.52 GHz. The CSRR-based MIMO antenna represents the reflectance of −12.2 dB at 9.05 GHz. It is observed that the proposed antenna provides channel independence and a lower coupling value.

### 4.4. Channel Capacity Loss (CCL)

**Figure 17:**
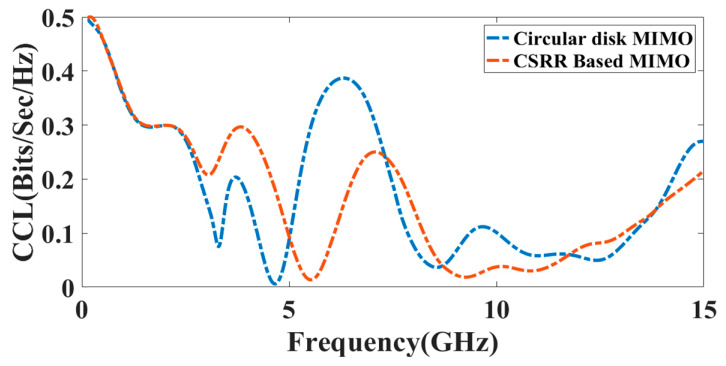
The CCL response of the presented MIMO antenna structure.

Correlation loss is a prime parameter for the CCL. The standard limit of CCL is less than 0.5 bps/Hz. The CCL response of the circular disk MIMO antenna is near zero at 4.95 GHz, and in the CSRR-based MIMO antenna, the CCL value is near zero at 5.08 GHz. Overall for the broad range up to 15 GHz, the CCL value is within the allowed range. Equations (5) and (6) are used to calculate the CCL. The CCL of the presented design is represented in Figure 17.

(5) $$C_{\mathrm{Loss}\;}=-\log_{2}\det\left[\alpha^{R}\right]$$

(6) $$\begin{array}{c} \left[\alpha^{R}\right]=\left[\begin{array}{cc} \alpha_{11} & \alpha_{12}\\ \alpha_{21} & \alpha_{22} \end{array}\right]\\ \alpha_{11}=1-({\left|S_{11}\right|}^{2}+{\left|S_{12}\right|}^{2}),\\ \alpha_{12}=S_{11}S_{12}+S_{21}S_{12},\\ \alpha_{21}=S_{22}S_{21}+S_{12}S_{21}\\ \alpha_{22}=1-({\left|S_{22}\right|}^{2}+{\left|S_{21}\right|}^{2}) \end{array}$$

The assessment of the two proposed and published works are presented in Table 2. The proposed CSRR metasurface design shows the highest isolation of 55 dB, and its overall complexity of the design is less than other available works.

## 5. Conclusions

Two metasurface designs (CSRR and circular disk) are proposed for designing a two-port MIMO antenna. The CSRR metasurface MIMO antenna design shows better reflectance and isolation than the circular disk MIMO antenna. The MIMO antenna design is optimized for variation in the inner ring diameter and outer ring diameter for the etched split ring resonator. The optimized value of the inner ring diameter is 6 mm, and the outer ring diameter is 8 mm. The ground region width also varied, and an optimized value of 8 mm width is obtained. The highest gain of 5.34 dB is obtained for the CSRR metasurface MIMO antenna design. The directivity of the CSRR metasurface MIMO antenna is higher than the other antenna. The comparison of different published methods also shows that the highest isolation of 55 dB is attained for the CSRR metasurface MIMO antenna. The performance measurement parameters were also analyzed for the proposed design in terms of *TARC*, *ECC*, *DG*, and CCL. The overall complexity of the design is also less than in other works. The design is applicable for C and X band applications.

## Figures and Tables

**Table 1:** Dimensions of presented design.

Length—35 mm	Width—25 mm	D—10 mm
DGW—8 mm	D1—8 mm	D2—6 mm

**Table 2:** Assessment of the presented MIMO designs with other MIMO structures.

Reference	Dimension (mm^2^)	Ports	Isolation (dB)	Complexity
Proposed design with CSRR metasurface	40 × 25	2	55	Less
Proposed design with circular disk metasurface	40 × 25	2	29	Less
[37]	30 × 15	2	35.8	Medium
[18]	48 × 31	4	21	More
[38]	20 × 20	2	24	Medium
[39]	19 × 19	4	35	More
[40]	31 × 31	8	16.1	More
[41]	35 × 30	4	17	Medium
[42]	12 × 51	4	25	Medium
[43]	34 × 21	2	22	Medium
[44]	23 × 13.5	2	19	Less
[45]	62 × 38	2	23	Medium
[46]	71 × 60	2	21	Medium
[47]	100 × 65	2	18	Less
[48]	43 × 38	2	15	Less
[2]	97.5 × 64	2	20	Medium
[49]	65.25 × 65.25	2	17	Medium
D				
D				

## Data Availability

The data will be made available at a reasonable request to the corresponding author.
